# Economic migration and the socio-economic impacts on the emigrant’s family: A case of Ward 8, Gweru Rural district, Zimbabwe

**DOI:** 10.4102/jamba.v10i1.414

**Published:** 2018-03-22

**Authors:** Everson Ndlovu, Richard Tigere

**Affiliations:** 1 Institute of Development Studies, National University of Science and Technology, Zimbabwe

## Abstract

Gweru Rural district in the Midlands province of Zimbabwe has witnessed an increasing number of outward migrations of breadwinners, leaving behind a desperate environment for families. This study was motivated by the realisation that most of the sick left behind, the elderly and children would visit the health centres unaccompanied, risking taking prescribed drugs incorrectly, thus further compromising their health. The study sought to establish the socio-economic effects of international migration on family members left behind in ward 8 of Gweru Rural. The study adopted a qualitative case study approach. Focus group discussions, questionnaires and structured individual interviews were used to elicit for data. Non-probability sampling design was used because of small samples available. Convenience and purposive sampling techniques were particularly used. Data were manually analysed and presented both qualitatively and quantitatively. The study revealed that international migration particularly to South Africa, especially by non- professionals, was not yielding the much expected economic gains; instead it was characterised by more negative social effects on the emigrant’s family. The study recommends that emigrants should consider migrating with their loved ones and, where it is not feasible, to put in place sound alternative caregiving arrangements. The study has provided an insight into international migration and its effects on left-behind families. However, a more comprehensive and quantitative survey remains critical to delving deeper into this migration phenomenon, particularly on how both the emigrant and left-behind spouses handle the issue of conjugal rights.

## Introduction

This study was conducted in Midlands province of Zimbabwe, in Gweru Rural district, Ward 8. Gweru is the sixth largest city located in Central Zimbabwe and is the administrative capital of the Midlands province. Ward 8 is located in Lower Gweru, which is by Zimbabwean standards a well-developed communal rural settlement, located about 40 km north-west of the city of Gweru. Lower Gweru stretches a further 50 km to the west. According to Mugandani et al. (2012), the greater part of Gweru lies in agro-ecological region 4, which is prone to droughts and the situation is compounded by climate change and variability. The average rainfall per annum in Lower Gweru is between 450 mm and 650 mm, poorly distributed and erratic. Such climatic conditions hardly support rain-fed agriculture-based livelihoods. The predominant farming system is subsistence farming, based on drought-tolerant crops such as sorghum, millet and rapoko. Farmers also grow some short-season maize varieties, groundnuts and cowpeas. De-industrialisation as a result of economic meltdown, high unemployment rates and limited commercial agricultural activities in this region have become the order of the day. The productive population often find themselves immigrating to South Africa especially and to other countries in the region and beyond to seek alternative livelihood opportunities. The city is also close to Beitbridge Border Post through which emigrants enter South Africa. South Africa is popularly known as ‘Egoli’ (the city of gold) and individuals who migrate to South Africa are nicknamed ‘injiva’ in Lower Gweru as they are known to bring ‘goodies’ when they next visit home. The ‘injiva’ are the envy of many young people in Lower Gweru and they act as their role models and whenever an opportunity arises, many youths would not hesitate to migrate to South Africa. Another reason why people from Lower Gweru flock to South Africa could be the use of common languages between South Africa and Zimbabwe. Ndebele and Zulu languages are similar and ethnic groupings in the two countries have very strong historical and cultural ties. The Ndebele people in Zimbabwe are said to have emigrated from South Africa long back. It was against this background that Gweru, and particularly Lower Gweru, was chosen for the study.

According to the ZIMSTAT (2012), Ward 8 consists of 912 households from 20 villages. The economic crisis in Zimbabwe that reached its peak during the 2007–2009 era, coupled with the prevailing negative political climate, motivated some Zimbabweans, particularly the productive age groups, to ‘flee’ their country to the west and neighbouring countries, mainly South Africa and Botswana, to seek alternative livelihood options. According to Kanyenze et al. (2011), the official rate of inflation in February 2009 had reached an alarming 231 million per cent and that made life unbearable in Zimbabwe. ZIMSTAT and IOM (2010) and IOM (2009) posit that there were 11 620 emigrants in 2005, mainly to other African countries. As they migrated, some left their loved ones behind, without adequate care and support.

One of the researchers of this study, a professional counsellor at Lower Gweru Rural Clinic, working with Doctors Without Borders in an HIV and AIDS Project, observed that the majority of women, children and old people would come to the clinic unaccompanied and some children and old patients used to default on treatment and thereby risking drug failure that could lead to full-blown AIDS and shorten their lives, because their parents and caregivers had migrated to other countries. The study sought to establish the socio-economic impact of international migration on left-behind families. It is anticipated that the findings could be used to minimise or reduce the negative effects of international migration on families remaining behind.

### Research objectives

This study had the following research objectives:

- to establish the socio-economic impact of international labour migration on families left behind
- to determine the effects of international labour migration by parents and guardians on the behavioural and educational outcomes of the children left behind
- to establish what could be done to avoid or minimise any negative effects of migration on the families left behind.

### Limitations of the study

The study used a qualitative case study research design; research findings of case studies can hardly be generalised to wider contexts. Research findings of this nature can hardly be generalised even to the whole Midlands province, in which this research was conducted, owing to smaller samples used. However, it acted as an eye-opener to transnational migration trends and its impacts on families left behind and would provide a basis for further study and policy formulation. Some emigrants from Ward 8, who had fled xenophobic attacks and violence in South Africa, were currently in Zimbabwe with their families. Probably because of great fear instilled in the affected emigrants and their families, the majority of research participants were reluctant to participate in the study for fear of victimisation. Creating an enabling environment was of paramount importance to ensure increased participation by respondents. Some research respondents, particularly school children and women, were suspicious of the study; they assumed that the research team could be spying on their migrant fathers and husbands. They feared that the worst could happen to their beloved ones. One of the students was overheard saying that, ‘if you participate in this study, your relatives in South Africa will be burnt alive’. This could have contributed to a small sample used in the study; therefore, the study only worked with those willing and consenting to participate. Issues of anonymity and confidentiality of their disclosures were emphasised and the consent of the respondents was sought after the participants were assured that the study was entirely for academic purposes. The participation of the local political and community leadership and school heads in the study worked to allay suspicions and misconceptions about the study.

### Literature review

International migration is a common phenomenon worldwide and people continue to migrate for various reasons and to various destinations. According to the immigration has become one of the most important issues in the contemporary global economy and it is further argued that over 110 million people now reside outside the county of their birth. These observations, among other reasons, have motivated this study. The aim was to understand more about this phenomenon and its socio-economic effects on the families left behind, with a focus on Gweru Rural district, Ward 8, in the Midlands province of Zimbabwe.

Theories underpinning the study are the neoclassical economic theory and the new economics of labour migration (Kurekova 2011). According to Borjas (1990) as cited in Castles (2000:2), the neoclassical economic theory holds that ‘the main cause of migration is the individual’s efforts to maximize their income by moving from low to high wage economies’. This is partly true considering the migration discourse in Zimbabwe. The majority of Zimbabweans and even other African citizens, particularly in southern region, tend to flock to South Africa to seek better livelihoods, because South Africa is perceived to be the most economically sound country in southern Africa. The new economics of labour migration (household model) was also found relevant to international migration discourse in Gweru Rural district. The household model shifts focus from an individual to a household as a unit of analysis. Decisions to migrate are not made by isolated individual actors, but by larger units such as families or households in which people act collectively not only to maximise expected income but also to minimise risks associated with various market failures (Stark 1984 cited in Massey et al. (1993). The United Nations Children’s Fund (UNICEF) (2007:3) asserts that ‘Migration is often considered a domestic survival strategy, in which one family member emigrates in order to guarantee the support for the whole family’. This is true in the Zimbabwean context. In the traditional Shona and Ndebele cultures, decisions that affect individual family members are made by the entire family as a social unit, including decisions to migrate. If an emigrant fails to send remittances back home, it would be considered a disservice to the entire family and there would be a family outcry.

Although the economic emigrants’ main intentions are to improve their economic prospects and those of their families left behind, in some instances that is not always possible. Schmalzbauer (2004) as cited in Mazzucato and Schans (2008) states that emigrants live in cramped, rundown apartments and skimp on meals and leisure goods in order to have the extra money to send home to family members. It is further argued that at times unrealistic expectations among those who stayed behind can lead to resentment on their part if they feel remittances are not sufficient. In some extreme cases, this mutual resentment resulted in a breakdown of families (Hashim 2006). Most migration studies conducted focus on the economic gains and very few focus on the social effects on family members left behind (Maphosa 2005; Thonge & Ncube 2014). Those that tried to establish the social effects of international migration on families left behind were mainly conducted in European and Asian continents and they tended to be culture insensitive and their findings could be irrelevant to Africa, and Zimbabwe in particular. Past migration studies in Zimbabwe tended to focus on the economic benefits of migration. This study concentrated on both economic and social effects of international migration on families left behind by mobile breadwinners.

Migration studies conducted in Albania and Bulgaria focused on both economic and social effects on left-behind families. According to Markova (2011), in Albania, more than half of the remittance recipient households lived in rural areas. It was further argued that remittances had been an important mechanism for poverty alleviation (Markova 2011). King et al. (2003), also cited in Markova (2011), concurred by arguing that access to emigration was seen by many as the only viable way out of poverty. The study also revealed that parental migration tended to have positive economic benefits to the children left behind. From the studies in Albania and Bulgaria, it is further revealed that international migration tends to have negative social effects on families left behind. Giannelli and Mangiavacchi (2010) argued that parental migration had a negative impact on children’s learning outcomes in Albania. School attendance was compromised by the absence of the parents. It was argued that lack of parental care could lead to relational and psychological problems in children in the long run. According to Markova (2011), many women were separated from their husbands, and just over 26 000 married women were living without their husbands at the time of the 2001 census. Studies in Nicaragua and Mexico found that children resented the absence of their fathers and some mothers reported increased parenting problems with their children, as these children tended to misbehave (UNICEF 2007).

According to UNCIEF (2007), the most serious problem concerning the elderly people is that they lose their family and social support. Guentcheva et al. (2003) as cited in Markova (2011) argue that there were high dropout school rates among children of migrant parents in Bulgaria. It was reported in other cases that children were left in the care of grandmothers or other relatives. They became easily spoiled and undisciplined, as they would not obey their elderly grandparents or other relatives serving as their guardians. These children would start smoking, drinking and eventually leave school altogether. According to HelpAge International and UNICEF (2010), parental involvement in migration results in major changes in obligations and responsibilities of grandparents. The grandparents assume the responsibility of educating and looking after the children. The additional obligations and responsibilities grandparents are subjected to increased physical, psychological and moral pressure (Share & Kerrins 2009).

According to Guentcheva et al. (2003) in Markova (2011), in the Albanian and Bulgarian studies, parental migration had more negative effects than positive effects on the children left behind. It is argued that teachers were of the view that children with emigrant parents often had cash sent by their parents and usually they become undisciplined. The study further postulates that children suffer too from the absence of their fathers (Cretella 2017). This is supported by Mazzucato et al. (2015) who argued that generally children living in transnational families have higher levels of psychological distress than those living with their parents at home. Children tend to suffer emotionally, especially if mothers migrate and leave them behind (Parrenas 2005 cited in Mazzucato et al. 2015). The study of the Filipina migrant domestic workers reveals that emotional suffering and feelings of abandonment of children left behind are common in cases where the mothers migrate (Mazzucato et al. 2015). It is further argued that to compensate for their absence, mothers would try to stay in close contact using phones and by sending money and goods (Howard 2011). Graham and Jordan (2011) also stated that children of migrant fathers in Indonesia were most likely to suffer from emotional disorders and those in Thailand were affected by conduct disorders, following the migration of their fathers.

Bradley and Corwny (2002) as cited in Graham and Jordan (2011) suggested that there was a close link between the socio-economic status of the children left behind and their mental health. Graham and Jordan (2011) proposed that left-behind children from wealthier households were less likely to suffer from emotional problems, as compared to their counterparts from poor households. The findings were based on the study with Indonesia and Philippine children. Wealth appears to be a protective factor in the dynamics of parental migration and children’s emotional suffering. According to the EuropeAid Project (2012), studies on Moldova and Georgia concluded that when parents decided to migrate, they would consider the effects of their decision on the children, as the central motive for their migration was improving the opportunities and the general welfare of their children. It was further argued that parents would only migrate if they could put in place acceptable caregiving mechanisms that included social safety nets. The study seemed to suggest that international labour migration could have insignificant negative effects on the welfare of the children left behind by their migrating parents in Moldova and Georgia, as transnational migration is carefully planned.

De Haas and Van Rooij’s (2010) study on rural Morocco revealed that international migration households enjoyed better living standards on average in terms of housing, sanitation, access to piped water and so on. Research findings further revealed that the proportion of extremely poor households was far higher among non-migrants. According to De Haas and Van Rooij (2010), international migration has negative effects on left-behind women. Findings established that more households were headed by women and that migration was generally seen as a major cause of the increasing number of female-headed households in Morocco, as spouses divorced. It was also revealed that all women living in migrant households experienced an increase in responsibilities as a consequence of migration. They had to take over most of the husband’s responsibilities and become responsible for managing almost all household affairs. De Haas and Van Rooij (2010) argued that this doubling of responsibilities was a burden and a source of conflict and divorce. The development impact of remittances in Chivi district, Zimbabwe, revealed that remittances from migrants in South Africa played an insignificant poverty reduction and developmental role and showed that very little asset accumulation was being done through remittance income (Thonje & Ncube 2014). The asset identified by households included cattle, goats, donkeys, scotch carts and tools, which were mostly not accumulated through remittance income and these were assets of little value.

According to Maphosa (2005), remittances from Zimbabwean migrants working in South Africa constituted a large proportion of households’ assets in Mangwe district. He further argued that remittances had a significant effect on community livelihoods in the southern parts of rural Zimbabwe. Stein (2003) as cited in Maphosa (2005) also asserted that although a large proportion of remittances was used to meet basic needs, these remittances contributed to improvement of the living standards of the households that received them. Remittances tended to have a positive effect on the development of households with a migrating family member. However, it was noted that these remittances had little development effect at community level.

These seemingly contradictory findings noted above seem to suggest that socio-economic effects of international migration could vary according to geographical locations. Therefore, this tends to justify the relevance of the current study that sought to establish socio-economic effects of international labour migration on the families left behind in Gweru Rural district, Ward 8, in Zimbabwe. The study could help to shed more light on this complex international migration phenomenon, because it is contextual and could act as tiebreaker to some of the results obtained from past studies that have been conducted elsewhere.

This study has looked at transnational migration and its socio-economic effects on the emigrants’ families and what could be done to avoid or minimise its negative effects (National Geographic Society 2005). However, the issues of intimate relationships and conjugal rights in migrant families were not adequately addressed by this study. A future qualitative study on this topic would be a welcome development to ensure all issues affecting families of migrant family members, including the breakdown of marriages, are fully addressed. While in foreign lands, emigrants experience a number of challenges. For example, emigrants particularly of the African origin have become victims of deadly xenophobic violence in South Africa. Therefore, the welfare of emigrants while in the host or receiving countries is also a possible area of enquiry.

## Research methodology

Permission to conduct the study was sought from responsible authorities: the District Education Officer, the District Administrator, the Chief, Ward 8 Councillor and the Officer Commanding Zimbabwe Republic Police Gweru Rural district. Once clearance was done by relevant government departments, the village heads then facilitated entry into villages. Written informed consent was obtained first and participants were assured of confidentiality of their disclosures.

A qualitative case study research design was used for this study. Individual face-to-face interviews, semi-structured questionnaires and focus group discussions were used to gather data from the research participants. Triangulation was used to validate data obtained. The study population consisted of all left-behind spouses, elderly people and children in Gweru Rural district, Ward 8. According to Zimbabwe National Statistics Agency (2012), Ward 8 consists of 20 villages and 912 households. A non-probability sampling technique was adopted, owing to small sample sizes available. Purposive and convenience sampling techniques were particularly used. Five out of the 20 villages were selected through convenience sampling. Only villages that were close to Makepese Rural Clinic were selected, as visits to the clinic also assisted in conveniently identifying and selecting research participants. Makepese Rural Clinic is the Community Meeting Place for Ward 8 villagers. The ward has a total of three secondary and four primary schools, respectively. Two secondary and two primary schools were conveniently selected to participate in the study because these schools had the highest numbers of children left behind by parents (District Schools Inspector 2015). Purposive sampling was also used to select teachers from the selected schools. From each selected school, three qualified senior teachers participated in the study. These high-profile teachers were chosen because of their seniority and assumed massive teaching and counselling experience. Only left-behind secondary school students who were willing participated in the study. Secondary school students participated in the study because the assumption was that such students, compared to primary school pupils, were more mature and had a more developed vocabulary that could enable them to appreciate issues being raised from the study and they could complete the questionnaires unaided. Secondary school children had the required cognitive and intellectual capacity to participate, to engage and interrogate the study. The school heads mobilised the students at their respective schools and they totalled 41 altogether. The interviews were conducted in small groups of students at each school to enable increased interaction and participation.

The ward councillor, with the help of five village heads, identified households with spouses left behind in the five villages. Out of the 48 individuals identified, 16 spouses were willing and thus participated in the study representing the five villages. Twenty-four left-behind elderly people from the five villages were conveniently selected as research participants. Two focus group discussions were conducted for the elderly people and participants were randomly classified into two groups of 12 participants each. The 16 spouses were individually interviewed and their responses were transcribed for further analysis and interpretation. Collected data were analysed manually using thematic approaches. Content analysis was carried out in order to identify the main themes that emerged from the interviews and focus group discussion responses. The responses were then categorised under themes to facilitate data analysis. Some direct citations verbatim were used to cement some narratives. This was consistent with qualitative research methodology that tends to produce narratives.

## Ethical considerations

Consent was sought from Ministry of Education, school authorities and local leadership to work with schools and communities.

## Findings and discussions

Spouse emigrants consisted of 87.5% males and 12.5% females and the majority (87.5%) of them were aged between 18 and 47 years (Table 1). Most women were left behind as their husbands migrated and it exposed spouses to HIV and Sexually Transmitted Infections (STIs) through infidelity. These results are similar to those produced by Markova (2011) in his study on Albania that revealed that many women were separated from their husbands. Spouses were denied their conjugal rights. Most emigrants in Ward 8 were sexually active and in the productive age group and it increased their chances of getting infected with HIV and/or AIDS and other STIs. Most left-behind women were overburdened with roles and responsibilities that their husbands used to do, like tilling the lands and herding cattle. Of the emigrants, 63% were not skilled and relied on menial jobs to earn a living. These emigrants could find it difficult to send meaningful remittances back home and their families could continue living in poverty. Only three emigrants managed to come back home within 3 and 6 months to enjoy their conjugal rights. On average, eight emigrants come back home once a year, assumedly during the festive season. Five emigrants exceeded a year without coming back home. All interviewed left-behind spouses indicated that they could not visit their emigrant spouses owing to financial challenges (Table 2).

**TABLE 1:** Emigrating spouses’ demographic data.

Demographic data	Number	%
**Gender**		
Male	14	87.5
Female	2	12.5
**Total**	**16**	**100.0**
**Age**		
18–47	14	87.5
48 and above	2	12.5
**Total**	**16**	**100.0**
**Occupation**		
Professional	6	37.5
Non-professional	10	62.5
**Total**	**16**	**100.0**

**TABLE 2:** Emigrants’ frequency of coming back home.

Period in months	Number of emigrants coming back home
3 months	1
4 months	1
6 months	1
12 months	8
18 months	1
24 months	1
40 months	1
60 months	1
264 months	1

One left-behind female spouse, a housewife research participant aged 37 years, had this to say on violation of her conjugal rights:

Yes, he sends good money. I have a good house and a nice car, but money can’t buy everything. My sexual and social life has been severely affected and my conjugal rights are being denied. It’s painful.

A male research participant, 47 years old, who does piece jobs in Ward 8 had this to say: ‘At first I managed to control these sexual feelings, but later I was overwhelmed, I now have a girlfriend, my painkiller’.

From the above discussion, it is evident that the majority of the interviewed spouses were of the view that they were being denied their conjugal rights and that was a great cause of concern for them. Only male spouses were honest and disclosed that they had girlfriends and they at times engaged in extramarital sexual activities. All 16 interviewed female spouses maintained that they remained faithful to their husbands. When asked how they manage their sexual drives, they gave various methods that however excluded infidelity. One of the female research respondents aged 35 years, a nurse-aid in the local clinic, had this to say about the issue of conjugal rights: ‘I pray when ever these sexual feelings haunt me’. Another housewife in her 40s said, ‘My sexual life is very dull, but I remain faithful, God gives the strength to resist the devil’. ‘Kana zvandikunda ndonamata,’ meaning ‘If I’m overwhelmed by these sexual feelings, I pray. I’m a Christian and above all, I don’t want to die of AIDS.’ This article argues that these female respondents above could actually be indulging in extramarital sexual encounters, but probably because of the traditional Shona and Ndebele cultures that condition married women to be faithful to their husbands, they could have given responses that they thought the study expected from them.

### Remittances and quality of lives of left-behind spouses and elderly persons

The majority of spouses and elderly people in the study indicated that their quality of lives never significantly changed owing to little remittances they receive. The third research participant, a housewife in her late 30s, had this to say about remittances she receives from her husband: ‘He sends R 200 after every 3 months and it’s not enough, what can I buy with R200?’ These findings are in contrast with those highlighted by Markova (2011) where he asserts that according to data released by the state Agency for the Bulgarians Abroad, at least 3000 people send amounts ranging between $100 and $300 to their families on a regular monthly basis. Stanchev et al. (2005) cited in Markova (2011) argue that remittances in Bulgaria have become an important element in improving the living standards of emigrant families and reviving the local economies through increased consumption and investment. Guentcheva et al. (2003) in Markova (2011) further confirm the use of remittances for consumption and purchase of houses and flats. However, in this study, the majority of the spouses believed that they continued to live in poverty, as put across by the forth respondent, a housewife, who works as a sales person in a local shop: ‘We are now worse off, the family sold goats for his transport and has he failed to replace the goats’. A younger housewife, still in her teens (another spouse) had this to say about her situation: ‘Nothing changed for the better except that I’m being denied my conjugal rights. I’m only 19 and he comes once in December, it’s not fair’.

Left-behind families continue to look to their migrated family members for their upkeep at home. Failure to send remittances back home is considered a disservice to the family left behind that allowed the emigrants to leave the country with the view of improving their economic prospects. This is consistent with the assumptions of the New Economics of Labour Migration (the household model). The left-behind spouses and the elderly tend to be overburdened with responsibilities and roles that were once done by the migrant spouses and older children.

Most of the elderly research participants were also of the opinion that the remittances they receive from their emigrant children were too little to meaningfully change their quality of life. They indicated that they were living from hand to mouth, as the money was not enough. Remittances could not buy anything big as they were too little. One of the respondents, a Ndebele-speaking elderly woman in her early 50s, said, ‘Kuyasifaka amastress, ungathengani nge US$20?’ [It causes stress, what can I buy with US$20?]. Another elderly participant had this to say about her migrant son:

Nothing has changed for the better. I am still the kind of person I was 10 years ago. I am still suffering. He abandoned his wife and kids and I am looking after them all.

A Shona-speaking old woman said, ‘Ndichiri kungogara muimba yangu yeuswa inoda kutodonha. Haatumire mari inokwana kuvaka imba yakanaka’ [I am still living in my dilapidated thatched hut that is about to fall. He does not send enough money to build a good house]. These research findings seem to suggest that left-behind families in Ward 8 are not benefitting much from the remittances received from their emigrant family members because it is too little. The left-behind families’ quality of lives has not significantly changed for the better.

### Remittances and quality of lives of left-behind students

**FIGURE 1:**
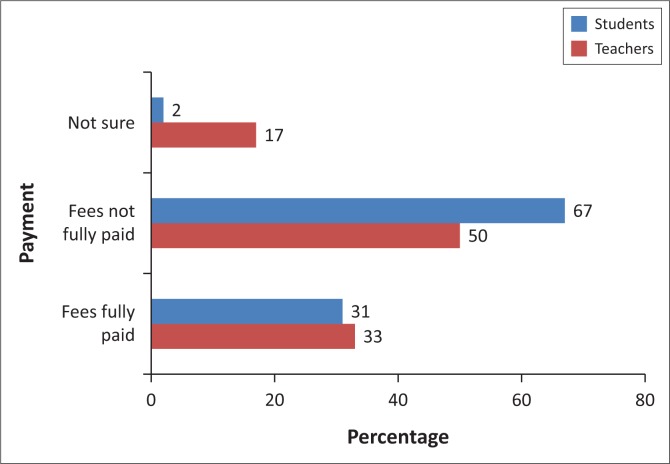
Parental migration and perception of payment of school fees and levies.

Results showed (Figure 1) that 50% of the teachers believed that students with migrating parents often do not pay school fees and developmental levies in full and on time. Similarly, the majority of students (67%) argued that their school fees and developmental levies were not being paid in full each school term. Left-behind students were not being provided for in terms of full school uniforms and stationary. These research findings are similar to those obtained by HelpAge International and UNICEF (2010) on Moldovan children. The study revealed that sometimes the caregivers use the money sent by the children’s parents to meet their own needs, instead of meeting the children’s needs. There was no significant change on the quality of lives of children left behind in terms of their economic and social well-being. From observations, it appeared that most of the students with migrant parents in Ward 8 were not better clothed in terms of full school uniform, as compared to their counterparts without migrant parents. Generally, the majority of students at schools visited had no full school uniforms. Either the remittances sent were not enough to meet all the students’ educational requirements or the caregivers might have been diverting the remittances for their personal use.

### Parental migration and behavioural patterns of left-behind children

**FIGURE 2:**
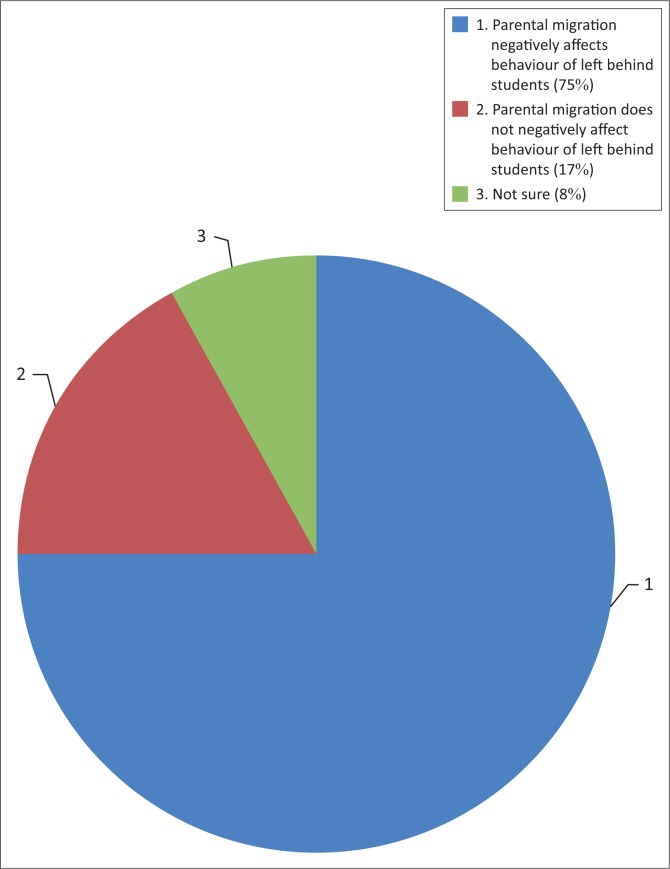
Perceived behaviour of left-behind students.

The majority of the teachers (75%) were of the opinion that children staying with their parents at home tend to behave much better than those with migrating parents because of close parental control and supervision at home (Figure 2). These findings are similar to those from UNICEF (2007) on studies carried out in Nicaragua and Mexico. The studies revealed that children resented the absence of their fathers and the left-behind mothers reported increased parenting problems with their children. Close parental control and supervision of children could have a positive effect on the overall behaviour of children. These research findings suggests that the majority of left-behind children in Ward 8 were undisciplined and they could have been engaging in delinquent activities such as drug and alcohol abuse because of lack of parental care and close supervision. This observation was made by teachers who complained that such children were always punished for various cases of indiscipline.

### Left-behind students and school discipline

**FIGURE 3:**
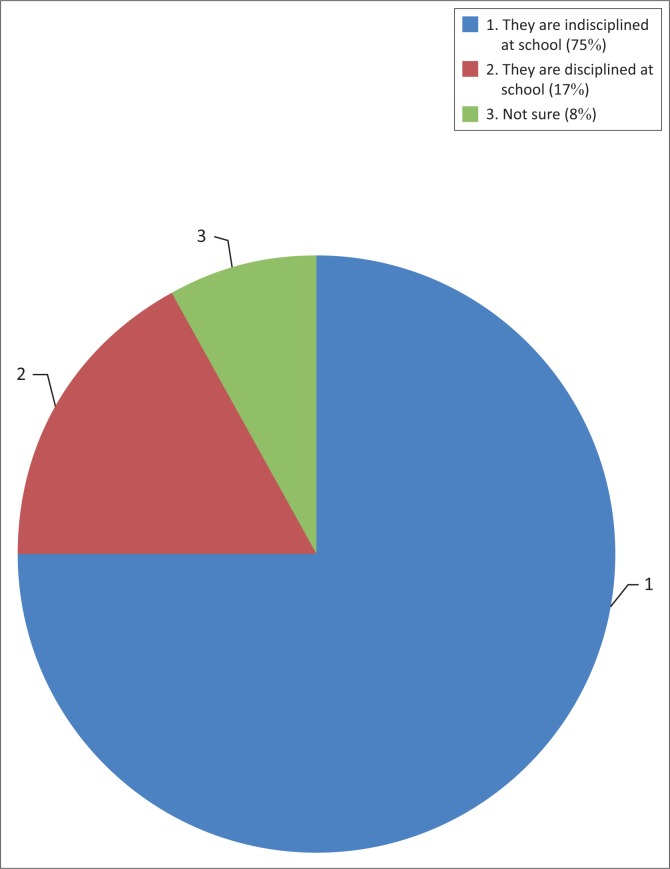
Left-behind students and discipline issues at school.

Results indicated (Figure 3) that the majority of the teacher informants (75%) believed that students whose parents migrated often misbehaved at school and are often in trouble with school authorities for various school offences. These children tend to lack close parental control and supervision at home and go on to exhibit such behavioural tendencies at school. Other research findings in this study show that the majority of these children stay with their grandparents who could no longer effectively control and supervise them because of old age. One of the teacher informants had this to say about left-behind students, ‘Children whose parents migrated tend to behave badly at school. They are the most rowdy, stubborn and unwilling to learn and very disrespectful’. According to teachers, other cases included absenteeism, bullying, stealing from other students and fighting with others. These results show that international parental migration tend to have undesirable effects on left-behind children in Ward 8, Gweru Rural district. It was reported by the interviewees that there was a perception that most of the left-behind children were smoking and abusing drugs and alcohol.

Guentcheva et al. (2003) as cited by Markova (2011) also revealed that there were high school dropout rates among children of migrant parents in Bulgaria. However, in this study, no effort was made to establish any link between parental migration and high school dropouts. Issues to do with higher rates of absenteeism and poor performance were however recorded as a cause for concern. It is further argued that these children became easily spoiled and undisciplined as they would not obey their elderly grandparents or other relatives serving as their guardians and would start smoking, drinking and, eventually, leaving school altogether (Markova 2011). Literature findings were similar to this study’s findings. Caregivers and teachers in this study revealed that children of migrant parents are often delinquent as they abuse drugs and/or alcohol and disrespect elders and teachers, probably because of lack of close parental control and supervision at home.

### Parental migration and left-behind children’s class performance

**FIGURE 4:**
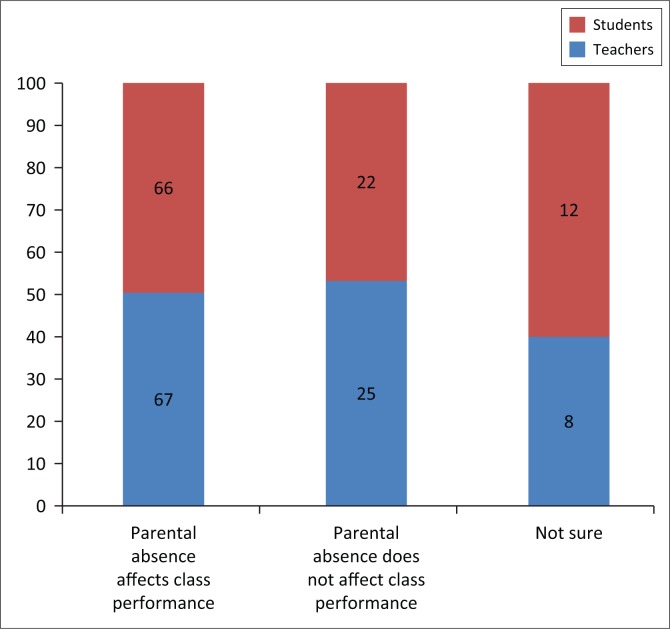
Parental migration and students’ class performance.

The majority of the teacher informants (67%) believed that students who stayed with their parents at home tended to perform better in class than those with migrant parents, because of close parental supervision at home (Figure 4). Of the students, 66% believed that their educational performance could have been much better had they were living with their parents at home. The fact that there could be no one at home to assist them to do their homework, coupled with lack of close parental control and discipline, could be the contributing factors to the poor class performance. The guardians they stayed with, particularly the grandparents, could fail to help these children with their school work, owing to high illiteracy rates among this special group. Their educational outcomes became severely compromised.

These research findings are consistent with the results obtained by Kandel and Kao (2000) as cited in Antman (2012) that suggest that children whose parents migrated tended to have lower educational aspirations than those with less exposure to migration as a pathway to mobility. The findings of this study concur with UNICEF (2008) findings that argue that, ‘Almost all children, care givers, teachers, and local authorities agree that the performance of children of migrants are significantly lower than those living with their parents’. These results suggest that children of emigrant parents in Ward 8 could be lagging behind in class performance and achievements as compared to their counterparts living with their parents at home.

## Conclusion and recommendations

### Summary of findings and conclusion

Massive exodus of Zimbabwean populace mainly to South Africa and Botswana during the period 2007 and 2009 could have been partly because of the economic meltdown and the prevailing negative political climate at that time. Indeed, Zimbabweans continue to cross the borders in search of better livelihoods even to date. However, it would seem that this emigration might be having little or insignificant economic and social impact on the families left behind. The study conducted by Thonge and Ncube (2014) in Masvingo province, in Chivi district of Zimbabwe, concluded similarly that there were very little economic gains of emigration, particularly to South Africa, on the families left behind. This study, unlike the one above which focused only on economic effects of emigration on families left behind, also looked at the social effects of emigration on the families left behind, including the issue of conjugal rights.

Findings revealed that there were little economic and social gains to the families left behind because the quality of their lives never significantly changed. The majority of those left behind continued to live in poverty. Interviewees argued that the remittances sent were not enough to meet the basic family needs and commodities. Teacher respondents were of the view that most students with emigrant parents did not pay school fees in full and timely and, in some instances, fees were hardly paid. Children with migrating parents were found to be performing below the standard of their counterparts living with their parents at home, probably because of lack of close parental control and guidance. Left-behind children were also found to be disrespectful and disobedient to teachers and elders they lived with. Left-behind spouses also complained about their conjugal rights being denied and violated. The majority of emigrants in the study were said to come back home once a year, usually for the festive seasons. Spouses revealed that they were sexually starved. The study argues that transnational emigration particularly to South Africa and Botswana was not yielding the much expected economic and social gains to the families left behind. In most instances, the quality of lives of the left-behind family members did not significantly change for better. The majority of these family members continue to live in poverty. Emigration appears to be having more negative socio-economic impacts than the desired benefits. Children with emigrant parents were reportedly engaging in delinquent activities and their educational performance was compromised. Left-behind families were not adequately cared for.

### Recommendations

To avoid or minimise the undesirable socio-economic impacts of international migration on left-behind family members, this study proposes a series of recommendation to be administered from a multi-sectoral approach at government, community and family levels.

#### At government level

The Zimbabwean government, through National Social Security Authority (NSSA), is encouraged to take the welfare of its retired elderly citizens seriously by giving monthly payouts that could enable them to lead decent lives. Currently, the payouts are too little and the elderly have no option other than looking upon their older migrating children as their insurance in old age and to be looked after. A robust old-age welfare security scheme is advised.

The Zimbabwean government should strive to put in place sound and consistent economic policies that would attract Direct Foreign Investment (DFI) to the country, with the view of developing the economy and creating employment for its people, in order to reduce or stop economic migration. Industrial regeneration is a must for the country and appropriate policy interventions and political reforms are a necessary springboard for a rebounding economy.

To improve the quality of lives of the abandoned elderly people, the government through the social welfare department should try to put in place mechanisms that aim at minimising the negative effects of transnational migration on abandoned elderly people. These may include committing the abandoned to old peoples’ homes. There is a need to engage the private sector through the private–public partnerships to allow the private sector to play its part in developing the economy. The ‘build-operate and transfer’ mechanism will motivate increased economic activity across all sectors. The private sector could also adopt public institutions like old people’s homes, schools and hospitals as part of the social responsibility drive.

#### At community level

Community-based organisations such as churches, where possible, should try to look after the abandoned elderly people and school children within their communities by providing them with basic needs like clothing, fees, food and psycho-social support services to improve their living standards and social well-being. The re-introduction of the community grain banks and related social safety nets through the extended family approach should be strengthened and capacity enhanced through deliberate capacity-building efforts by community-based organisations, community leadership and local government structures.

#### At family level

Where possible, spouses are encouraged to migrate together to safeguard their marriages and sexual reproductive health. Where this is not possible, migrating spouses should strive to visit their spouses they left back home more often to enjoy their conjugal rights and guard against cases of infidelity that could endanger their lives. This could, however, be negated by financial challenges. Awareness campaigns to this end will assist family members not only in terms of knowledge and in acquiring travel documents to ensure spouses regularly visit each other. Village leadership and local schools through the new curriculum could foster such teachings at family level.

Migrant parents are encouraged to consider migrating together with their children to safeguard the safety, social and emotional well-being of their children. Where this will not be feasible, an attempt could be made to decide on a sound alternative caregiving arrangement before they leave their children behind. If the parents can afford it, they should consider enrolling their children at a boarding facility provided parents regularly visit their children during scheduled times. Responsible and reliable extended family members, such as aunties and uncles, could be identified and entrusted with the caring of the left-behind children provided they are also assisted and empowered to do so.
